# TREATMENT OF BENIGN LYTIC BONE LESIONS WITH 45S5 BIOACTIVE GLASS: A CASE SERIES

**DOI:** 10.1590/1413-785220263404e304880

**Published:** 2026-07-17

**Authors:** Jaime Gomes Nogueira, Marco de Andrade Bianchi, André Ferrari de França Camargo, André Mathias Baptista, Olavo Pires de Camargo

**Affiliations:** 1 Universidade de Sao Paulo, Faculdade de Medicina, Hospital das Clinicas, Instituto de Ortopedia e Traumatologia IOT-HCFMUSP, Sao Paulo, SP, Brazil.

**Keywords:** Bone Neoplasms, Osseointegration, Biocompatible Materials, Orthopedic Surgery, Neoplasias Ósseas, Osseointegração, Materiais Biocompatíveis, Cirurgia Ortopédica

## Abstract

**Objective:**

To report a series of 9 cases of patients with benign cavitary tumor lesions who underwent intralesional resection (curettage) followed by cavity filling with 45S5 bioactive glass (Aktibone).

**Methods:**

This is a therapeutic case series study with level IV evidence. Patients diagnosed with benign lytic bone lesions who had a surgical indication for curettage followed by cavity filling with bioactive glass were included. In the postoperative period, patients underwent imaging examinations, which were qualitatively evaluated by a radiologist who determined the radiological diagnosis and the quality of osseointegration. The presence of imaging abnormalities was also assessed, and statistical analysis was performed.

**Results:**

Nine patients were included, 7 males and 2 females. The mean age was 25.8 years. The initial diagnoses varied, with enchondromas being the most frequent. The mean follow-up time after surgery was 8.4 months. All patients underwent preoperative radiographs. Most patients showed good clinical progression and good to partial osseointegration.

**Conclusion:**

The results reinforce that 45S5 bioactive glass demonstrates good performance as a bone substitute in benign lytic lesions, providing adequate osseointegration and a low complication rate. ***Level of Evidence IV; Case series.***

## RESUMO

**Objetivo:**

Relatar uma série de 9 casos de pacientes com lesões tumorais cavitárias benignas submetidos a ressecção intralesional (curetagem) e posterior preenchimento com biovidro 45S5 (Aktibone).

**Métodos:**

É um estudo terapêutico do tipo série de casos com nível de evidência IV. Foram incluídos pacientes com diagnóstico de lesão óssea lítica benigna e que tinham indicação cirúrgica de curetagem da lesão e posterior preenchimento da cavidade com o biovidro. No pós-operatório, os pacientes realizaram exames de imagem que foram avaliados de forma qualitativa por um médico radiologista que definiu o diagnóstico radiológico e a qualidade da osseointegração. Também foi observada a presença de anormalidades na imagem e foi realizada análise estatística.

**Resultados:**

Foram incluídos 9 pacientes, sendo 7 homens e 2 mulheres. A média de idade foi 25,8 anos. Os diagnósticos iniciais foram variados, com predominância de encondromas. O tempo de acompanhamento pelo serviço após a realização da cirurgia teve média de 8,4 meses. Todos os pacientes realizaram RX pré-operatório. A maioria dos pacientes tiveram boa evolução e osteointegração boa e parcial.

**Conclusão:**

Os resultados obtidos reforçam que o biovidro 45S5 apresenta bom desempenho como substituto ósseo em lesões líticas benignas, proporcionando adequada osteointegração e baixo índice de complicações. ***Nível de Evidência IV; Série de casos.***

## INTRODUCTION

The prevalence of benign bone lesions of low aggressiveness remains poorly documented, mainly due to their often asymptomatic presentation and insidious evolution, often being detected incidentally during complementary exams requested for the investigation of fractures or other musculoskeletal conditions.^1^ Among the main etiologies, enchondroma, osteoma, non-ossifying fibroma, and unicameral bone cysts stand out, which, although benign, may require surgical treatment to prevent pathological fractures and restore function.^1,2^

The most commonly employed surgical approach in these cases is curettage, whose objective is the complete removal of the pathological tissue while preserving as much healthy bone as possible.^3^ Subsequently, filling the resulting cavity is a fundamental step to restore mechanical strength and promote bone regeneration. Thus, autologous bone grafts are traditionally considered the gold standard due to their excellent osteogenicity, osteoinductivity, and osteoconductivity; however, they have significant limitations, such as morbidity of the donor site, pain, and limited availability.^3,4^ Alternatively, allografts are widely used, but they carry risks of pathogen transmission, immune reactions, and biomechanical failures. Other options include xenografts, bone cement, and synthetic bone substitutes, which vary in biological properties, mechanical properties, and cost-effectiveness.^3^

In light of this, among synthetic substitutes, the development of bioactive glasses marked a milestone in biomaterials engineering, being the first compounds capable of establishing a direct chemical bond with bone tissue.^5^ The bioglass exhibits controlled degradation and the release of bioactive ions, such as soluble silica, calcium, sodium, and potassium, which stimulate cell proliferation, bone neoformation, and angiogenesis, through the crystallization of apatite on its surface and modulation of the local microenvironment. The classic formulation 45S5, commercially known as Bioglass®, composed of 46.1% SiO_2_, 26.9% CaO, 24.4% Na_2_O, and 2.6% P_2_O_5_, demonstrates strong integration with the bone matrix, such that its removal is unfeasible without fracturing the adjacent bone after complete remodeling, which occurs between 6 and 18 months post-operation.^2,5–7^

In addition to its osteoconductive and osteoinductive properties, active bioglass exhibits an intrinsic antimicrobial effect, related to the increase in pH and osmolarity at the implanted site, making it unfavorable for bacterial growth.^5^ It is a safe, non-toxic, and chemically stable biomaterial that combines regenerative potential with infection prevention, positioning itself as a promising alternative in the management of low-aggressiveness lytic bone lesions.^6,8^

In this context, recent comparative trials and randomized studies reinforce that bioactive glass 45S5 graft substitutes provide outcomes similar to those of allografts in terms of lesion recurrence, need for reoperation, and functional scores in heterogeneous series of benign tumors.^2,9,10^ However, there are limitations in the global literature regarding factors such as lesion heterogeneity and relatively short follow-up, which impose caution in generalization, in addition to highlighting the need for multicenter studies with prolonged follow-up and long-term remodeling assessment.^2^

In this context, the evaluation of active bioglass 45S5 in bone lesions through imaging exams is essential to monitor the incorporation of the material, bone neoformation, and structural stability over time. Computed tomography (CT) has been widely used to quantify bone mineral density and monitor graft remodeling, allowing differentiation between residual material and regenerated bone.^10^ Magnetic resonance imaging (MRI), in turn, provides information about tissue integration and potential complications, such as tumor recurrence or inflammatory reaction, without exposure to ionizing radiation.^9^

Furthermore, recent studies highlight the role of serial radiography as an accessible and effective method for monitoring clinical evolution, although with sensitivity limitations compared to CT.^2^ Therefore, the combination of different imaging modalities has been recommended to provide a more comprehensive analysis of osteointegration and the safety of bioglass 45S5, ensuring greater diagnostic accuracy and support for clinical decision-making.^6^ Thus, the objective of this study is to report a series of 9 cases of patients with low-aggressiveness cavitary tumor lesions who underwent intralesional resection (curettage) and subsequent filling with bioglass 45S5 (Aktibone).

## MATERIALS AND METHODS

This is an observational, descriptive, and prospective study of case series. This is a therapeutic study with a level of evidence IV. The present research was conducted following the ethical standards established by the Nuremberg Code (1947), the Declaration of Helsinki (2000), and the Research Standards Involving Human Beings established by Resolution 466/12 of the National Health Council. This study was approved by the Research Ethics Committee (CEP) of IOT-HC-FMUSP, under opinion CAAE 58963622.3.0000.0068. The evaluated patients signed the Informed Consent Form (ICF).

The work was conducted by the Orthopedic Oncology Group of the Institute of Orthopedics and Traumatology of the Hospital das Clínicas of the Universidade de São Paulo (IOT-HCFMUSP). The surgeries were performed at the Surgical Center of the Institution.

## MATERIALS

The bioactive glass granules (size between 500-1000 μm), commercially named Aktibone, produced by Noraker Bioglass Company, Lyon, France, were fully donated by RCL Implantes, the official distributor of the product in Brazil, imported by Visão Implantes.

The material, designated as bioglass 45S5, is composed of 45% SiO2, 24.5% Na2O, 24.5% CaO, and 6% P2O5.

### Inclusion criteria

Patients enrolled at IOT-HCFMUSP with a diagnosis of low-aggressiveness lytic bone lesions, such as enchondromas, non-ossifying fibromas, and simple bone cysts, were included. Furthermore, the patients had surgical indications for various reasons, such as risk of fracture or orthopedic complications, pain, an increase in the size of the lesion, and risk of oncological progression.

### Data collection

The selected patients followed the usual routine flow of the Orthopedic Oncology Group.

The surgeries were performed according to good medical practice and with the established surgical techniques already in use by the Orthopedic Oncology Group. The bone lesions were accessed through a bone window created in the cortex using chisels; after curettage of the lesion, the internal walls of the lesion were smoothed with a high-speed drill, thus reducing the risk of residual neoplastic cells remaining in small recesses or spaces created by the microtrabecular bone; after this smoothing, the internal walls were cauterized with an electric scalpel. These measures are important for reducing the risk of local recurrence. Next, the cavity was filled with bioglass, and, whenever possible, the removed bone window was replaced to occlude the opening. The decision to add a prophylactic metal implant (plate and screws) depended on the anatomical region and dimensions of the lesion, and was made on a case-by-case basis.

The immediate postoperative procedures were performed as routinely done by the Group (prophylactic antibiotic therapy for 24 hours, dressing care, drains, motor physiotherapy, etc.). After discharge, the patients returned at intervals of 1, 2, and 4 weeks for evaluation of soft tissue healing and any acute complications; and at 3, 6, and 12 months for evaluation of consolidation, osteointegration, functional recovery, and any late complications. For this evaluation, imaging tests were used, such as X-ray, computed tomography, and magnetic resonance imaging.

### Data analysis and statistics

In the postoperative evaluation, the patients underwent imaging tests that were qualitatively assessed by a radiologist from the service. After a summative analysis of all the tests performed by each patient, the radiological diagnosis was defined as: good evolution, poor evolution, or inconclusive. Additionally, regarding osseointegration, it could be classified as: good and total, good and partial, moderate, poor, and inconclusive. Abnormalities such as osteolysis, expansive calcifications, deformities, osteoporosis, and fractures, among others, were also observed.

Descriptive statistics of the quantitative data were performed: mean, median, maximum, and minimum values for each parameter.

### Risk analysis

Considering that bioglass 45S5 (Aktibone) is already a product widely used in the Brazilian market in various clinical situations) with no reports of complications, the risk of this research was considered low.

## RESULTS

Nineteen eligible patients were selected according to the inclusion criteria; however, ten were excluded during the research, with one patient due to a postoperative complication of fracture and loss of bone alignment, while the others (nine) lost follow-up after surgery. Thus, nine patients with low-aggressiveness lytic bone disease were included in the study, consisting of seven men and two women. Regarding age, the youngest patient was seven years old, and the oldest was fifty-four years old, with an average age of 25.8 years and a median of 22 years. The initial diagnosis was varied and included: Six enchondromas, two non-ossifying fibromas, and one simple bone cyst. The location of the lesions was diverse and is better described in Table 1.

**Table 1:** Location and diagnosis of bone lesions.

Patient	Diagnosis	Location of the lesion
A	Non-ossifying fibroma	Right femur
B	Enchondroma	Proximal phalanx of the right fifth digit
C	Enchondroma	Right femur
D	Simple bone cyst	Left humerus
E	Enchondroma	Right third metacarpal
F	Enchondroma	Distal phalanx of the right first digit
G	Non-ossifying fibroma	Right distal tibia
H	Enchondroma	Middle phalanx of the right fourth digit
I	Enchondroma	Proximal phalanx of the right fifth digit

Source: author, 2025.

Regarding the follow-up time by the service after the surgery, the average was 8.4 months, and the median was 8 months, with a minimum of 2 months and a maximum of 13 months. All patients underwent pre-operative X-rays. In the post-operative period, the average number of imaging exams performed by patients was 4.7 and the median was 5, with the minimum value corresponding to the patient who underwent 2 exams and the maximum to the patient who underwent 7 exams. The radiologist made qualitative observations of the lesions, which are better described in Table 2. Thus, it is observed that the majority of patients (6) had good evolution and good and partial osteointegration (3).

**Table 2:** Qualitative and summative assessment of radiological imaging exams..

Patient	Evolution	Osteointegration	Number of imaging exams performed	Types of imaging exams performed	Follow-up time	Observations
A	Inconclusive	Inconclusive	2	RX	2 months	Presence of expansive calcifications
B	Poor	Moderate	7	RX, TC, RM	13 months	Presence of osteolysis in about 70% of the area + fracture and deformity
C	Good	Good and partial	7	RX, TC, RM	13 months	—
D	Good	Good and total	5	RX, TC	8 months	Evolution with osteoporosis of the limb
E	Poor	Poor	5	RX, TC, RM	13 months	Progression of cartilage injury + fracture + Presence of a halo of osteolysis around the bioglass + No closure of the dorsal cortex
F	Good	Good and total	2	RX	2 months	Presence of a thin halo of osteolysis in the distal portion
G	Good	Moderate	5	RX, TC, RM	8 months	Presence of a halo of osteolysis around the bioglass + No integration with the adjacent bone marrow + No closure of the anterior cortex
H	Good	Good and partial	7	RX, TC, RM	11 months	Evolution with re-ossification
I	Good	Good and partial	3	RX	6 months	Presence of partial resorption on the medial face of the middle third

Source: author, 2025.

## DISCUSSION

The present study evaluated the use of bioglass 45S5 in the treatment of low-aggressiveness lytic bone lesions, including nine patients, predominantly male (77.7%), with an average age of 25.8 years. The lesions were classified as benign or non-aggressive behavior, including enchondromas, non-ossifying fibromas, and simple bone cysts. The majority of patients (66.7%) showed good clinical and radiographic evolution, with signs of satisfactory osteointegration over an average follow-up of 8.4 months, demonstrating the osteoconductive and osteoinductive potential of bioglass 45S5 in cavitary bone defects.

These findings are consistent with recent evidence from the literature confirming the potential of bioglass 45S5 as an effective and safe bone substitute. Nogueira et al. (2024) highlight that 45S5 exhibits high bioactivity, with ionic release of calcium, phosphorus, and silicon, promoting the formation of a carbonated hydroxyapatite layer that favors osteointegration.^6^ This characteristic stimulates osteoblastic differentiation and accelerates the bone regeneration process, making the material a promising alternative to autologous grafts, which still represent the gold standard but with limitations associated with donor site morbidity.^6^

Moreover, clinical studies also corroborate the good response of bioglass in benign bone lesions. Thus, Samade et al. (2022), in a series of pediatric cases, observed good incorporation of the material and absence of major complications in cavity defects treated with bioglass 45S5 after curettage of benign bone tumors.^11^ The average consolidation time was similar to that observed with autologous grafts, with a lower risk of recurrence and no need for surgical reintervention. Similarly, Ma et al. (2021), when comparing the use of bioglass 45S5 with allogenic bone grafts in symptomatic lesions of the calcaneus, reported equivalent clinical results, with radiographic evidence of progressive bone formation and good integration of the biomaterial.^10^

In terms of clinical applicability, bioglass 45S5 presents significant advantages, such as excellent biocompatibility, absence of immunogenic response, and ease of intraoperative molding. Recent experimental studies also demonstrate that the incorporation of therapeutic ions, such as copper or strontium, can enhance the osteogenic and antimicrobial effects of 45S5, expanding its clinical application possibilities.^12^

In the present study, the presence of three cases with partial osteointegration of enchondromas may be related to the location of the lesions, in the femur and phalanges, or to the volume of the treated defect, factors already pointed out by Jin, Neuville, and Brauer (2025) as determinants in the biomechanical performance of bioglasses, since the amount of 45S5 applied directly influences the regeneration process and the mechanical strength of the newly formed bone.^12^ Additionally, individual differences in bone metabolism, age, and quality of the receptor bed may also interfere with the biological response to the biomaterial.^12^

Another relevant aspect is the short average follow-up time (8.4 months) observed in this study. Although the initial results are encouraging, the literature indicates that complete replacement of bioglass with mature bone tissue may take up to 18 to 24 months.^6,11^ Thus, prolonged follow-up is essential to confirm structural stability and absence of recurrence of the lesions, especially in tumors with more active behavior. Such evolution, however, is hindered by the loss of follow-up of patients in the orthopedic service.

Regarding the choice of the best imaging exam to evaluate the osteointegration of bioglass in the postoperative period, it was noted that computed tomography is more suitable as it provides better visualization of the lesion, in accordance with Tsukayama et al. (1999).^4^ However, due to the more difficult access to CT and the higher radiation used, serial X-rays may be used for monitoring in cases of good evolution and good osteointegration, opting for CT only in complicated cases or those that do not show good evolution on X-ray. Such analysis is consistent with the study by Incesoy et al. (2025), in which radiographs were performed at different times (6 weeks, 3, 6, 12, and 24 months) to monitor bone filling in the postoperative period and with Giavaresi et al. (2008), in their randomized prospective study with 12 patients where imaging studies were performed with serial simple radiographs, in addition to computed tomography, magnetic resonance imaging, bone scintigraphy, and SPECT.^2,13^

On the other hand, it should be considered that the small sample size and the absence of a control group limit the extrapolation of the results. The diagnostic and anatomical heterogeneity of the lesions also constitutes a bias factor, making it difficult to standardize the response to treatment. Despite these limitations, the findings are consistent with recent literature and suggest that bioglass 45S5 represents a safe and effective alternative for the treatment of bone defects resulting from benign lytic lesions, especially in young patients with good bone quality.

## CONCLUSION

Therefore, the results obtained in this series of cases reinforce that bioglass 45S5 performs well as a bone substitute in low-aggressiveness lytic lesions, providing adequate osteointegration and a low complication rate. However, studies with larger samples, longer follow-up, and objective analysis of bone regeneration are necessary to consolidate its role in the surgical management of these lesions.

## Data Availability

The underlying contents of the research text are contained in the manuscript.
